# Counseling on injectable contraception and HIV risk: Evaluation of a pilot intervention in Tanzania

**DOI:** 10.1371/journal.pone.0231070

**Published:** 2020-04-03

**Authors:** Janine Barden-O’Fallon, Jennifer Mason, Emmanuel Tluway, Gideon Kwesigabo, Egidius Kamanyi

**Affiliations:** 1 Carolina Population Center, MEASURE Evaluation, University of North Carolina at Chapel Hill, Chapel Hill, North Carolina, United States of America; 2 Department of Maternal & Child Health, Gillings School of Global Public Health, University of North Carolina at Chapel Hill, Chapel Hill, North Carolina, United States of America; 3 United States Agency for International Development, Washington, D.C., United States of America; 4 United States Agency for International Development, Dar es Salaam, Tanzania; 5 Department of Epidemiology and Biostatistics, Muhimbili University of Health and Allied Sciences, Dar es Salaam, Tanzania; 6 Department of Sociology, University of Dar es Salaam, Dar es Salaam, Tanzania; KHANA, CAMBODIA

## Abstract

In a context of high rates of HIV prevalence, concerns over hormonal contraceptive use and the potential for increased risk of HIV acquisition have led to increased attention to counseling messages, particularly for users of the injectable. However, the consequence of adding additional HIV risk messages to family planning counseling sessions was not well understood. This evaluation assessed the effect of providing revised injectable and HIV risk counseling messages on contraceptive knowledge and behavior during a three month pilot intervention. The pilot intervention was conducted September-November 2018 with all eligible family planning clients in ten healthcare facilities located in the Iringa and Njombe regions of Tanzania. Data collection for the evaluation occurred November-December 2018 and included 471 client exit interviews, 26 healthcare provider interviews, and the extraction of service statistics for 12 months prior to the intervention and three months of the intervention. Univariate and bivariate analyses were used to assess quantitative interview data. Thematic qualitative assessment was used to assess qualitative interview data from healthcare providers. Interrupted time series analysis was used to assess changes in the trend of contraceptive uptake. Results indicate that the counseling messages did not cause a decrease in the uptake of injectables (Depo-Provera): 97 percent of interviewed clients received Depo-Provera at their visit; sixty percent reported an intention to use condoms for dual protection. The analysis of service statistics showed no statistical difference in the trend of Depo-Provera uptake between the pre-intervention and intervention periods (p = 0.116). Overall knowledge of counseling messages by clients was good; however only 64.8% of women correctly responded that women at risk of getting HIV can use any method of family planning. Providers’ knowledge of the messages was high, though it appears that not all messages were consistently provided during the counseling sessions. The findings from this evaluation provide evidence that complex HIV counseling messages can be implemented in family planning programs in Tanzania, and potentially in other countries that are considering how to better integrate HIV risk messages into family planning counseling.

## Introduction

### Background

In 2016, an updated systematic literature review was conducted in response to a growing body of evidence showing a possible increase in the risk of HIV acquisition associated with the use of hormonal methods, specifically depot medroxyprogesterone acetate (DMPA), a progestogen-only formulation of the injectable [1]. The review found that if the relationship between DMPA and HIV acquisition risk was causal, there would be up to a 50 percent increased risk of HIV acquisition for women using DMPA (hazards ratio of 1.5 or less) [1]. The Evidence for Contraceptive Options and HIV Outcomes (ECHO) Trial, a large multicenter, randomized trial comparing HIV incidence between users of DMPA, copper IUD (Cu-IUD), and a levonorgesterel implant, was conducted in Eswatini, Kenya, South Africa, and Zambia. The study was designed to address whether there is an increased risk of HIV acquisition when comparing women using DMPA with Cu-IUD, DMPA with levonorgestrel implant, and Cu-IUD with levonorgesterel implant. The results from the trial were published in mid-2019, and show that there is not an increased risk of HIV incidence among users of DMPA as compared to users of Cu-IUD or the implant [2]. The findings support the continued use of these methods in areas of high incidence of HIV. However, the overall HIV incidence in the study population was 3.8 per 100 woman-years, which is “alarmingly high”, and signals a need for strengthened family planning (FP)/HIV programming [2].

In 2017, guidance from the World Health Organization (WHO) classified progestogen-only injectables as “Category 2,” meaning the advantages of using the method generally outweighed the disadvantages for women at high risk of HIV, but that additional counseling was warranted [3]. Based on available evidence at the time, the 2017 WHO Guidance Statement recommended efforts to strengthen communication and counseling on injectable use and HIV risk (referred to as hormonal contraceptive- HIV [HC-HIV] counseling), especially in settings where women are at high risk of acquiring HIV. Tanzania, with an estimated adult HIV prevalence rate of 4.6 (5.7 for adult women), an HIV incidence rate of 2.5 per 1,000 adults, and with injectables being the most popular method of contraception (accounting for 37.0 percent of modern method use in 2015–16) is a country where counseling on HIV acquisition risk is relevant for the general population of FP clients [4,5].

### Description of the pilot intervention

A pilot intervention of HC-HIV counseling messages was conducted in Tanzania from September 1- November 30, 2018 to test outcomes of implementing the 2017 WHO Guidance Statement on (1) uptake of hormonal contraceptives, particularly Depo-Provera; (2) reported intention to use condoms as a dual method; (3) client’s level of knowledge and understanding of messaging related to HC-HIV risk and (4) provider’s knowledge of and attitudes about the delivered messages. The pilot intervention involved a number of organizations and projects, including the Tanzania Ministry of Health, Community Development, Gender, Elderly, and Children (MOHCDGEC); United States Agency for International Development (USAID)/Tanzania; USAID/Washington offices of HIV/AIDS and Population and Reproductive Health; and USAID-funded projects including Boresha Afya Southern Zone, Breakthrough ACTION, and MEASURE Evaluation. The intervention took place in Iringa and Njombe, two southern regions of Tanzania. These regions were selected due to the relatively high level of modern contraceptive use (32.0 percent in Iringa and 45.0 percent in Njombe) and HIV prevalence in the adult population (11.3 percent in Iringa and 11.4% in Njombe) compared to the national levels [6,7]. The intervention was implemented in ten primary healthcare facilities with relatively high numbers of Depo-Provera uptake (Depo-Provera was the only formulation of the injectable available in Tanzania at the time). The five selected health facilities from Iringa were Mafinga Hospital, Ilula Hospital, Ipogolo Health Center, Kimande Health Center, and Ihongole Health Center. The five selected health facilities from Njombe were Makete Hospital, Kibena Regional Referral Hospital, Makambako Hospital, Njombe Health Center, and Ipelele Health Center. All women attending these health facilities during the intervention period who were (1) currently using Depo-Provera or (2) expressed interest in using Depo-Provera were eligible to receive the counseling on the risk of HIV acquisition. A mix of long-acting and short-acting methods of contraception were available to clients throughout the intervention, including in addition to Depo-Provera, oral contraceptive pills, implants, Cu-IUD, and male and female condoms.

Prior to implementation, context-appropriate counseling messages and materials were developed. The counseling messages were based on a review of the Strategic Communication Framework for Hormonal Contraceptive Methods and Potential HIV-related Risks and the FP Global Handbook for providers [8,9]. The context-appropriate messages were approved by stakeholders at a launch meeting in January 2018 [S1 Table]. They can be summarized as follows: (1) Depo-Provera may increase the risk of acquiring HIV; (2) It is not known if Depo-Provera causes a higher risk of HIV; (3) Other contraceptive methods (such as oral contraceptive pills and implants) do not appear to increase the risk of HIV; (4) Women at risk of HIV can still use any method of FP; and (5) Women at risk of HIV who are using Depo-Provera should also use condoms. Breakthrough ACTION, a USAID-funded project led by the Johns Hopkins Center for Communications Programs, in collaboration with the Tanzania MOHCDGEC, led the development and pretesting of counseling materials. The following materials were developed and produced in English and Swahili: (1) A flip-chart page entitled, “Use of Depo-Provera and Risk of HIV Infection” to be added to the regular flip-chart used during FP counseling sessions; (2) A booklet entitled, “Hormonal Contraception and HIV: Frequently Asked Questions and Counselling Messages”; (3) An amendment to an already existing wall chart that updated the section on “Depo-Provera”; and (4) A provider reminder sticker [10]. The counseling messages and materials were reviewed and adapted at a stakeholder meeting, convened in April 2018 by the MOHCDGEC.

Orientation of healthcare providers from the selected health facilities to the issue of potential HC-HIV acquisition risk and training on the counseling messages and materials also occurred before the start of the pilot intervention. The training followed a training-of-trainers model used by the Tanzanian MOHCDGEC for refresher and technical update trainings. After orientation to the counseling messages, master trainers practiced explaining the new messages to providers as well as to provide the counseling messages themselves. The master trainers also determined the optimal time to provide the messages during a counseling session was when advantages and disadvantages of Depo-Provera are explained. The master trainers then led a two-day rollout training to 49 healthcare providers. The providers were supplied with the counseling support materials for their facilities. They were advised to not take any leave or transfers during the three-month implementation and to conduct the counseling themselves—i.e., not to train others at their facilities to provide the messages. Upon leaving the training, on August 29, 2018, the healthcare providers were told to initiate the pilot intervention, and to continue providing the messages to every eligible client until November 30, 2018.

Implementation of the intervention was monitored to ensure consistent and accurate transmission of the counseling messages. At each visit, one or more providers were observed during FP counseling sessions and a monitoring checklist was completed. The checklists verified whether providers were communicating the counseling messages according to the standards set at the provider training. Items on the checklist included whether clients were counseled on the possible increased risk for HIV while using Depo-Provera; whether condoms were presented as a means of protection from HIV while using Depo-Provera (i.e., as dual method) and women were counseled on their use; whether clients were free to choose any method, including Depo-Provera; and whether clients’ opinions, concerns, and reactions were respected and correctly addressed by the provider following the delivery of the HC-HIV acquisition risk messages.

## Materials & methods

The evaluation was led by MEASURE Evaluation, a USAID funded project headed by the Carolina Population Center at the University of North Carolina at Chapel Hill. The evaluation used a mixed-methods approach that included multiple types of data—client exit interviews, provider interviews, routine service statistics, and routine monitoring of provider-client counseling—to provide a comprehensive understanding of the impact of the HC-HIV counseling messages. Data collection was conducted by MEASURE Evaluation in collaboration with Health and Development International Consultants (HDIC), a sub-contractor based in Dar-es-Salaam, Tanzania. The data were collected in November and December 2018.

Client’s demographic characteristics, knowledge of the communication messages, intention to use condoms, and FP method chosen during the appointment were assessed through a short client exit interview utilizing a structured interview tool with both closed- and open-ended questions. Exit interviews were conducted with women exposed to the counseling messages in a private location at the healthcare facility. Women were eligible for a client exit interview if they (1) attended a FP appointment at one of the ten healthcare facilities involved in the intervention during the period of data collection, and (2) received the counseling messages. Eligible women included continuing Depo-Provera users and new Depo-Provera users, as well as women who received the counseling messages but then selected an alternative method. Eligible women were of reproductive age (ages 15–49). Ineligible women included clients who did not consider Depo-Provera use, who were known to be HIV positive, or who for other reasons did not receive the counseling. During periods of data collection, providers were instructed to give a green colored card to clients who received the counseling and a yellow colored card to clients who did not. In this way, data collectors were able to identify women eligible for the interview without needing to collect information on HIV status as a screening question or as part of the interview. All women with green colored cards were approached for interview and agreed to be interviewed. A minimum sample size of 422 client exit interviews was derived by setting an acceptable level of significance of p<0.05, a power of 80 percent to detect significant change in contraceptive method uptake, and an additional 10% to account for incomplete surveys or other potential data quality issues. According to a review of service statistics for the 12-month period of March 2017 to February 2018, the number of Depo-Provera clients seen per week ranged from 8–37 clients in the ten selected facilities. As a result, the number of clients interviewed at each facility varied according to client volume. Consecutive clients were approached for interviews until samples were reached.

Provider feedback on the messages, knowledge, and level of comfort providing the messages, was assessed through structured key informant interviews with healthcare providers. All eligible providers from each of the participating facilities were targeted for interview (30≥n≥20). Eligible providers were those who (1) received the training on HC-HIV counseling messages, (2) participated in the intervention, and (3) were available for the interview during one of the days of data collection.

In addition to information on contraceptive use collected through client exit interviews, service statistics on uptake of Depo-Provera, other hormonal contraceptives (oral contraceptive pill and implant), and total FP methods (Depo-Provera, oral contraceptive pill, implant, Cu-IUD and condom) were collected from each facility participating in the intervention. There were a total of 15 months of service statistics: 12 months prior to the intervention and three months of the intervention (September 2017 through November 2018).

### Ethics approval

The evaluation proposal and draft data collection tools were reviewed and approved by the Tanzania National Institute for Medical Research on July 20, 2018 and the University of North Carolina Institutional Review Board on August 2, 2018. Research clearances and permits were also obtained from the Tanzania Commission for Science and Technology, the Tanzania National Bureau of Statistics, and the President’s Office, Regional Administration and Local Government. Additionally, permission to conduct the research in the selected health facilities was obtained from regional and district authorities prior to data collection. The study is registered at ClinicalTrials.gov, identifier NCT04160169. There were no technical deviations to the approved protocol; however, a delay in the initiation of the intervention led to a two-month postponement in data collection. A protocol deviation tracking log was also completed due to the higher than expected number of completed interviews.

Informed written consent and assent was obtained from all study participants. The need for parental consent of minors was waived by the Tanzania and University of North Carolina ethics review boards. The consent form for healthcare providers explained the use of audiotapes. All data was deidentified. There were no personal benefits to participating in the study, other than the opportunity to participate in the research process and provide input on FP counseling and services. There were no anticipated risks to clients or providers to participate in the evaluation. Providers were assured that their names and identifying data would not be used in the analysis or presentation of findings. Quality assurance protocols were followed to ensure data protection, data quality, and confidentiality throughout the study.

### Analysis

At the client level, outcomes of interest were method uptake, including intention to use condoms as a dual method, and knowledge of the HC-HIV counseling messages. Univariate and bivariate statistics were used to assess these data. Select outcomes were assessed by demographic variables, mainly age (categorized in five- year age groups). The Pearson Chi-square test was used to examine equivalence of distribution between demographic variables and outcomes of interest where appropriate. At the provider level, univariate statistics were used to present quantitative measures of knowledge of the HC-HIV counseling messages, feedback on the training, and use of counseling materials.

Qualitative interviews with healthcare providers were transcribed and translated from Swahili to English by the local research partner. Analysis of the interview data involved three iterative steps: reading, organizing and displaying, and reducing. First, each transcript was read at least twice to begin to identify preliminary themes. Sections of transcripts were highlighted to help bookmark quotes that were potentially meaningful or unexpected. Next, to organize and display the data, a matrix was developed to summarize typical and atypical responses to interview questions, as well as additional information or perspectives provided by the providers. The final process of reducing the data involved identifying themes and sub-themes from the data, and assessing whether there were meaningful differences by region.

For the analysis of service statistics, interrupted time series (ITS) analysis using segmented regression was used to assess the overall impact of the counseling messages on contraceptive uptake among FP clientele in participating health facilities. The routine data from the ten intervention facilities were aggregated to the total number of FP clients seen per month and total number of clients seen per month for Depo-Provera, pill, and implant. Separate models were run for each outcome variable. ITS commands using ordinary least-squares regression were run using STATA version 14 [11]. Newey-West standard errors were used to adjust for autocorrelation. The Cumby-Huizinga test was used to assess autocorrelation and to correctly fit the model. According to the test, autocorrelation was not convincingly present in any of the models, up to the five lags tested. Default adjustments for autocorrelation (lag[0]) were therefore used in the models. For each outcome variable, a line plot of the predicted variables was combined with a scatterplot of the actual values over time.

## Results

### Findings from client exit interviews

**Fig 1:**
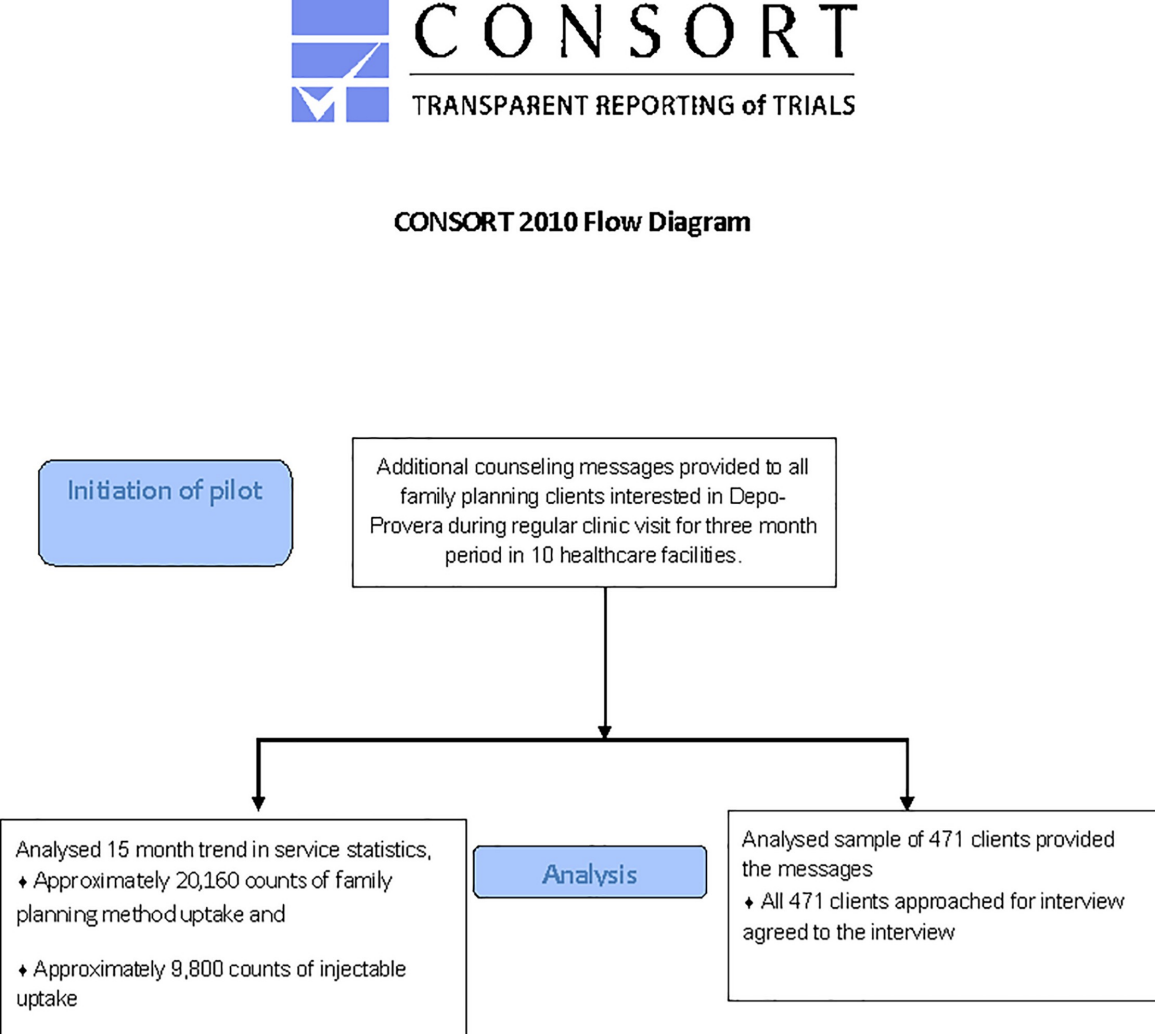
CONSORT diagram.

A total of 471 client exit interviews were conducted with women receiving the HC-HIV counseling messages. The CONSORT diagram is shown in Fig 1. Demographic characteristics of the interviewed clients are shown in Table 1. The age of the interviewed clients ranged from 17 to 49 years, while the mean age was 27.9 years [SD = 5.9]. About 30 percent of the women interviewed had a secondary education or higher. Almost all women interviewed had at least one child, with 60.3 percent having either one or two children. Ninety percent of the clients were either married or living with their partner.

**Table 1:** Background characteristics of FP clients percent distribution of interviewed women by age group, education, number of living children, and marital status, Tanzania 2018.

Age (years)	%
17–19	5.1
20–24	23.6
25–29	35.2
30–34	22.5
35+	13.6
**Education**	
No education	6.4
Primary	63.5
Secondary or higher	29.9
No response	0.2
**Number of living children**	
No children	2.5
1 child	26.3
2 children	34.0
3 children	20.0
4+ children	17.2
**Marital status**	
Never in union	6.4
Married/living together	89.8
Separated/divorced/widowed	3.4
No response	0.4
**Number of women**	**471**

Prior to the FP visit, 68 percent of interviewed women were using a modern contraceptive method. The most common methods used were Depo-Provera (57.7 percent) and implant (4.9 percent). Dual methods, condoms plus any other method, were used by 13.6 percent of the women. Method use varied somewhat by age: adolescents were the least likely to be using any modern method prior to the visit (45.8 percent), while women age 35 and over were the most likely to have been using a modern method at the time of the clinic visit (84.4 percent) (results not shown in table).

Table 2 shows the pattern of method continuation and switching in the female clients after their visit. At the clinic visit, 97 percent of clients initially opting for Depo-Provera received Depo-Provera after the counseling, while 1.5 percent received the implant, 1.1 percent received the pill, and 0.2 percent received the Cu-IUD. One client decided to stop using a contraceptive method in order to become pregnant.

**Table 2:** Method continuation and switching at visit percent distribution of the change in contraceptive use as a result of the clinic visit, by age group, Tanzania 2018.

Age	Continued Depo- Provera (%)	Started Depo- Provera after non-use (%)	Switched to Depo-Provera from a different Method (%)	Started another method after non- use (%)	Switched from Depo- Provera to a different method (%)	Stopped using a method (%)	Number of women
17–19	41.7	54.2	4.2	0.0	0.0	0.0	24
20–24	49.5	36.9	11.7	0.9	0.9	0.0	111
25–29	50.0	34.3	11.4	1.2	2.4	0.6	166
30–34	63.2	22.6	12.3	1.9	0.0	0.0	106
35+	76.6	14.1	4.7	1.6	3.1	0.0	64
**Overall**	**56.1**	**30.6**	**10.4**	**1.3**	**1.5**	**0.2**	**471**

During the FP visit, 97.2 percent of clients reported that HIV risks were discussed, 91.3 percent reported that dual-method protection was discussed, and 85.8 percent of clients reported that the provider discussed STIs. As a result of the counseling, 60.3 percent of clients reported that they plan to use condoms as a method of STI/HIV prevention, though the proportion varied significantly by age from 53.1 percent of clients age 35 and over to 75 percent of adolescent clients (Table 3).

**Table 3:** Intention to use condoms percentage of women ages 17–49 who report they plan to use condoms for protection against STIs, including HIV, by age group, Tanzania 2018.

Plan to use condoms as method of STI/HIV protection	Age*						
	17–19 (%)	20–24 (%)	25–29 (%)	30–34 (%)	35+ (%)	Overall (%)	N (Total = 471)
Yes	75.0	71.2	57.8	53.8	53.1	**60.3**	**284**
No	25.0	28.8	38.0	40.6	45.3	**36.7**	**173**
Don’t know	0.0	0.0	4.2	5.7	1.6	**3.0**	**14**

*p-value<0.05

Clients were asked to summarize, in their own words, what they remembered from their visit about the risks associated with Depo-Provera use and HIV acquisition. The interviewers used no prompts. The most common responses reflected a correct understanding of the HC-HIV counseling messages, though less than half of all women mentioned any single one of the messages. Table 4 shows that the most commonly mentioned messages were that women at risk of HIV and who are using Depo-Provera should use condoms (48 percent) and that Depo-Provera may increase the risk of HIV (40.1 percent). Overall, fewer than five percent of women reported an incorrect understanding of the messages, that Depo-Provera can cause STIs/HIV (3.8 percent), though the percentage was somewhat higher for women ages 35 and over (7.8 percent). Responses did not differ significantly by age group.

**Table 4:** Spontaneous mention of messages related to Depo-Provera and HIV acquisition percentage of women 17–49 who spontaneously recalled various messages from counseling on Depo-Provera and potential increased risk of HIV acquisition, by age group, Tanzania 2018.

	Age					
Message	15–19 (%)	20–24 (%)	25–29 (%)	30–34 (%)	35+ (%)	Overall (%)
Women at risk of HIV who are using Depo-Provera should also use condoms	29.2	47.7	48.2	45.3	59.4	**48.0**
Depo-Provera may increase the risk of HIV	29.2	44.1	40.4	36.8	42.2	**40.1**
It is not known if Depo- Provera causes higher risk of HIV	25.0	20.7	22.9	22.6	28.1	**23.1**
Depo-Provera use does not protect against STIs/HIV	16.7	13.5	16.3	17.9	10.9	**15.3**
Women at risk of HIV can still use Depo-Provera	12.5	13.5	9.6	13.2	10.9	**11.7**
There are other long-acting and effective methods of FP	12.5	12.6	7.8	13.2	12.5	**11.0**
Don't know/remember any messages	20.8	9.9	10.2	8.5	4.7	**9.6**
Depo-Provera use can cause STIs/HIV	4.2	1.8	3.6	3.8	7.8	**3.8**
Number of women	24	111	166	106	64	**471**

However, a significant difference in the distribution of responses by education level is seen for the spontaneous mention of the message that Depo-Provera may increase the risk of HIV. Women completing secondary education or higher were the most likely to mention this statement (54.6 percent), followed by women completing primary education (35.5 percent), while women with no education were the least likely to mention this statement (16.7 percent) (results not shown in table). The mention of other messages did not vary significantly by education level.

A number of true/false statements related to the use of hormonal contraceptive methods were read to the interviewed clients to assess their knowledge of the HC-HIV counseling messages. Table 5 shows the distribution of clients correctly answering the eleven true/false questions. The statements are presented in the order they were given to the clients. The number of correct responses ranged from zero (one client answered “don’t know” to all statements) to eleven, with the mean number of correct statements at 7.5 per client. More than 90 percent of women were able to correctly answer three of the statements; another statement on dual-method use was correctly answered by 80.3 percent of the interviewed clients. Overall, more than half of women were able to answer correctly all the statements except two.

**Table 5:** Client’s knowledge of HC-HIV counseling messages: True/false percent distribution of knowledge about hormonal contraceptive methods and associated risk of HIV acquisition among women ages 17–49, N = 471, Tanzania 2018.

True/false statement		Correct (%)	Incorrect (%)	“Don’t know” (%)
1.	Hormonal contraceptives, such as implants, pills, and Depo-Provera, are very effective in preventing unintended pregnancy when used consistently and correctly. (TRUE)	94.1*	1.7	4.2
2.	Hormonal contraceptives, such as implants, pills, and Depo- Provera, are very effective in preventing STIs when used consistently and correctly. (FALSE)	76.4	14.0	9.6
3.	Dual-method use, using a condom with another FP method, will help prevent both unintended pregnancy and HIV/STIs. (TRUE)	80.3*	14.4	5.3
4.	Using Depo-Provera may increase a woman’s risk of getting HIV. (TRUE)	67.5	20.6	11.9
5.	Using implants may increase a woman’s risk of getting HIV. (FALSE)	25.3	49.0	25.7
6.	Taking contraceptive pills can increase a woman’s risk of getting HIV. (FALSE)	28.9	51.2	20.0
7.	Women at risk of getting HIV can use any methods of FP. (TRUE)	64.8	18.5	16.8
8.	Women who think they are at risk of getting an STI or HIV should use condoms. (TRUE)	93.6	2.1	4.2
9.	Women who think they are at risk of getting HIV and are using Depo-Provera should also use condoms. (TRUE)	91.7	4.0	4.2
10.	Some research has found that women who use Depo- Provera and are exposed to HIV are slightly more likely than other women to get an HIV infection. (TRUE)	66.5*	15.7	17.8
11.	We do not know whether or not Depo-Provera c*auses* higher risk of HIV. (TRUE)	56.7	20.4	22.9

*p-value<0.05

Knowledge of the HC-HIV counseling messages was mostly consistent across age groups. In a comparison of the distribution of correct answers by age group, only three statements had significant variance at a p-value of <0.05, as indicated in Table 5. For the statement on the effectiveness of hormonal contraception at preventing unintended pregnancy, the percent of women answering correctly ranged from a low of 88.3 percent for women ages 20–24 to a high of 100 percent for women ages 17–19 and ages 35 and over. For the statement on dual-method use for protection against unintended pregnancy and acquisition of STIs and HIV, the percent of women answering correctly ranged from 75.7 percent for women ages 20–24 to 92.2 percent for women ages 35 and over. Finally, for the counseling message related to the research on HC-HIV acquisition risk, the percent of women answering correctly ranged from 50 percent of the adolescents ages 17–19 to 79.7 percent of the women ages 35 and over.

### Findings from healthcare provider interviews

Twenty-six interviews (fourteen in Iringa and twelve in Njombe) were conducted with healthcare providers trained to provide the HC-HIV counseling messages. Twenty-three of the providers were female and three were male. The providers were nurses, including nurse mid-wives, and clinical officers. The level of experience of the providers ranged from less than one year to 27 years of experience, with an average of 8.7 years. Twelve providers had five or fewer years of experience, six providers had between six and ten years, and eight providers had more than 11 years of experience.

Two techniques were used to assess providers’ knowledge of the HC-HIV counseling messages. Providers were first asked to summarize the HC-HIV counseling messages in their own words. Interviewers marked each of the messages that were correctly mentioned. No prompts were used during the interview. Table 6 shows the distribution of providers correctly mentioning each of the five key counseling messages. Two of the five key messages were mentioned by more than 80 percent of the providers; that Depo-Provera may increase the risk of HIV and that women at risk of HIV who are using Depo-Provera should also use condoms. One of the messages, “other contraceptive do not appear to increase the risk of HIV” was rarely mentioned by the providers interviewed. Only two providers spontaneously mentioned all five of the messages, while 74 percent of providers (n = 19) mentioned at least three of the messages.

**Table 6:** Providers’ knowledge of HC-HIV counseling messages: Spontaneous response number and percent distribution of knowledge of HC-HIV counseling messages among twenty-six trained healthcare providers, Tanzania 2018.

**Counseling statements**	**Mentioned by providers Number (%)**
Depo-Provera may increase the risk of HIV.	21 (81)
It is not known if Depo-Provera causes higher risk of HIV.	19 (73)
Other contraceptives (such as pills and implants) do not appear to increase the risk of HIV.	3 (12)
Women at risk of HIV can still use any method of FP.	12 (46)
Women at risk of HIV who are using Depo-Provera should also use condoms.	22 (85)
**Number of statements correctly mentioned**	
One	1 (4)
Two	6 (23)
Three	14 (54)
Four	3 (12)
Five	2 (8)

Providers were then asked to answer a number of true or false questions about the counseling messages. Table 7 shows the distribution of providers correctly answering the twelve true or false questions. All of the providers correctly answered seven of the statements, and more than 80 percent of providers correctly answered ten of the statements. Only two statements showed a mixed level of knowledge among providers: the statement that using Depo-Provera may increase a woman’s risk of getting HIV and the statement that taking contraceptive pills can increase a woman’s risk of getting HIV.

**Table 7:** Providers’ knowledge of HC/HIV counseling messages: True/false number and percent distribution of correct knowledge of HC-HIV counseling messages among twenty-six trained healthcare providers, Tanzania 2018.

True/False Statement		Correctly answered Number (%)
1.	Hormonal contraceptives, such as implants, pills, and Depo-Provera, are very effective in preventing unintended pregnancy when used consistently and correctly. (TRUE)	25 (96)
2.	Hormonal contraceptives, such as implants, pills, and Depo-Provera, are very effective in preventing STIs when used consistently and correctly. (FALSE)	26 (100)
3.	Dual-method use, using a condom with another FP method, will help prevent both unintended pregnancy and HIV/STIs. (TRUE)	26 (100)
4.	Using Depo-Provera may increase a woman’s risk of getting HIV. (TRUE)	13 (50)
5.	Using implants may increase a woman’s risk of getting HIV. (FALSE)	22 (85)
6.	Taking contraceptive pills can increase a woman’s risk of getting HIV. (FALSE)	19 (73)
7.	Women at risk of getting HIV can use any methods of FP. (TRUE)	24 (92)
8.	Women who think they are at risk of getting an STI or HIV should use condoms. (TRUE)	26 (100)
9.	Women who think they are at risk of getting HIV and are using Depo- Provera should also use condoms. (TRUE)	26 (100)
10.	Some research has found that women who use Depo-Provera and are exposed to HIV are slightly more likely than other women to get an HIV infection. (TRUE)	26 (100)
11.	We do not know whether or not Depo-Provera c*auses* higher risk of HIV. (TRUE)	26 (100)
12.	All women, regardless of HIV status, have the right to choose the number, timing, and spacing of their pregnancies. (TRUE)	26 (100)

During the interviews, all providers initially stated they were confident they understood WHO’s guidance, with some indicating they were “100 percent” confident. Many service providers exemplified their high understanding of the WHO counseling message through sharing how they counseled their clients. In responding to the types of questions clients ask after receiving the counseling message, one service provider stated:

“*They ask if injections cause HIV infection*. *We respond by telling them it is not true*, *but a particular person’s habit is the one that triggers the infections*. *Injections do not cause HIV infections*.*”* —Provider in Njombe

However, a few providers were not clear on why use of dual methods was being emphasized for women using Depo-Provera and not for women using other methods of hormonal contraception. These providers stated that emphasizing use of dual methods for women on Depo-Provera made it seem that the practice was not as relevant for users of other methods.

With regard to communicating the HC-HIV counseling messages with clients, typical FP counseling was reported to take anywhere from 10 to 30 minutes, and providers reported that the HC-HIV counseling messages, which took about two to five minutes within the overall counseling time, did not take too much time to deliver. Almost all healthcare providers stated that when they first communicated the HC-HIV counseling messages, their clients were initially concerned that Depo-Provera caused HIV. Some clients were specifically concerned that the Depo-Provera injection itself contained HIV, and that the virus was intentionally placed in the injection by a party external to the clinic or service provider.

“*They* [clients] *tend to believe that the manufacturers insert HIV in such methods*. *But we explain to them that they don’t have HIV infections because they have been certified by experts*.*”—*Provider in Iringa

All service providers who counseled these clients assured them that Depo-Provera does not include HIV, and many continued their counseling by explaining that HIV infection is a result of behaviors such as having multiple partners and having sex without a condom. Most healthcare providers were confident that after further clarification clients understood that Depo-Provera does not have HIV, but they differed somewhat in how well they thought clients understood the messages by the end of the counseling session. Some providers thought their clients had a good understanding of the messages, while others believed their clients did not fully understand what they were being counseled on.

*“It is difficult since as I said*, *you may ask if she has any question…but she does not*. *You ask her a question but she does not answer*. *It is difficult to know if they understood what you told her*. *Most of the time very few acknowledge the fact that they did not understand*.*”*—Provider in Njombe

Service providers in Iringa were generally more forthcoming about their clients’ confusion around the counseling message. In clarifying the part of the counseling message that was challenging for clients to understand, one provider shared:

*“The* [part of the] *message [that is confusing is] for those using injections who have the possibility of being infected*. *It is the difficult message to explain to the customer*. *It is hard to understand*.*”* —Provider in Iringa

One strategy used by a few providers to both gage whether clients understood the counseling message and, in the process, help clients better understand the message, was to ask clients to repeat the message back, and then follow-up with clarifications, if needed, or questions. These providers were clear that they believed merely telling the message to the client was not effective counseling, and that instead, they needed to have a discussion with the client.

A few providers shared that clients wanted to understand the exact mechanism through which Depo-Provera might increase their risk of HIV. These providers expressed frustration that the potential pathway is unknown and that they were unable to share definitive information about the risk of using Depo-Provera or the manner in which Depo-Provera might increase HIV risk with their clients. Providers shared that either none or very few clients who were using Depo-Provera at the time of the clinic visit switched to a different method. When asked whether clients who switched did so because of the new counseling message, the providers stated that they were unsure of the reason.

The healthcare providers offered suggestions on how to improve the counseling messages and tools. One provider thought that the counseling messages should first include an explanation about how Depo- Provera works to prevent pregnancy, and also the risk factors of HIV:

“*They should improve on the part of how to give a message to the customer on how Depo-Provera is working and how the customer may be infected with HIV*. *Depo-Provera does not cause HIV infection but HIV is caused by this and that; but if you use a condom you will not get infected…”—*Provider in Iringa

Several providers suggested that improving the manner of delivery would make the counseling messages more effective. One provider stated that practicing communicating the message in a conversant manner, rather than reading them from the flip charts, would help clients understand the message. (This practice was encouraged during the training.) Two providers suggested first establishing rapport with the client at the beginning of the session, and then asking her questions to ensure she understands. Another provider recommended emphasizing that all users of hormonal contraceptives should use dual methods. Finally, one provider specifically recommended using the following sentence:

“*Risky behavior can lead an injection method user to be infected with HIV*.*”—*Provider in Njombe

### Findings from service statistics

**Fig 2:**
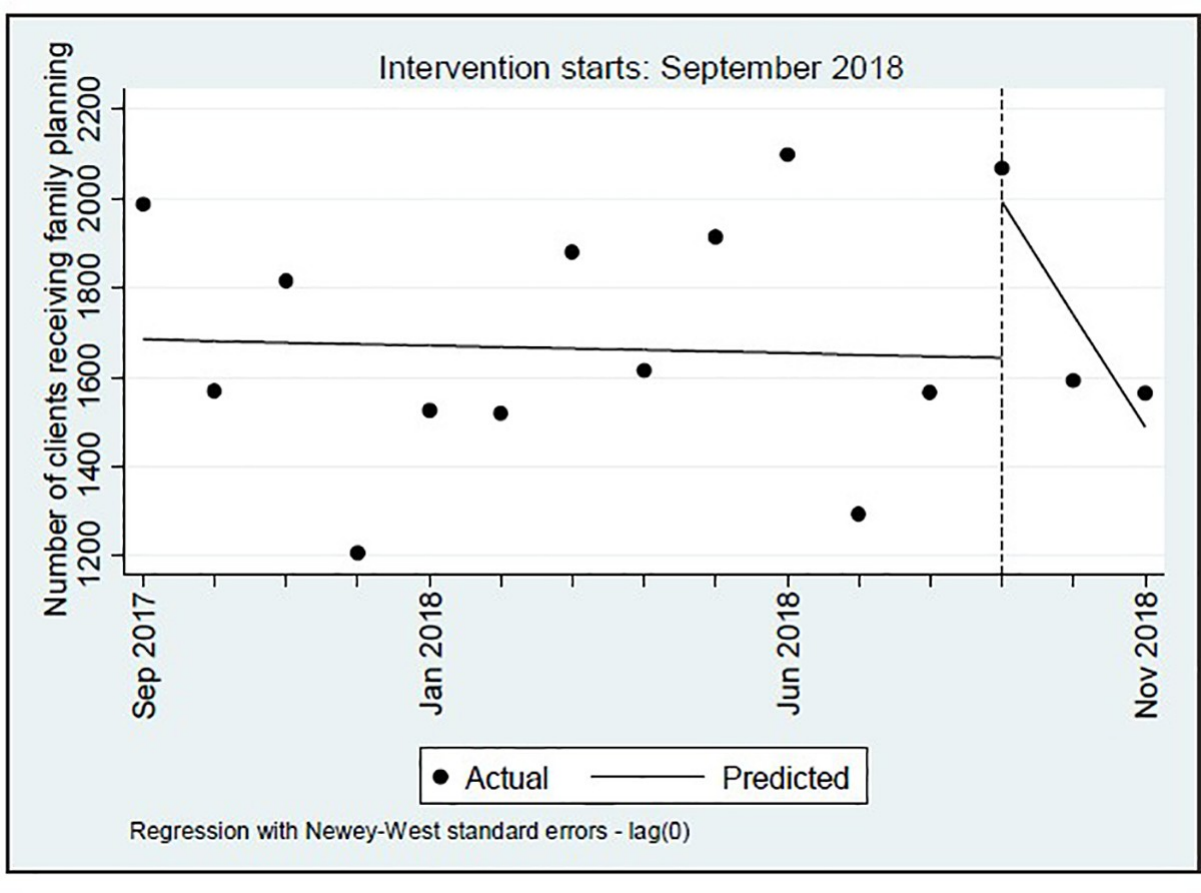
Graph of total number of FP clients per month in the ten pilot intervention facilities from September 2017 through November 2018, Tanzania 2018.

During the 15-month period of study, the lowest monthly number of FP clients seen at the ten facilities was 1,206 and the highest monthly total was 2,097; the mean number was 1,680 [SD = 268]. The ITS regression found a significant decrease in the monthly trend of FP clients of 248.6 clients for the intervention period as compared to the pre-intervention period (P = 0.003, CI = [-396.0,-101.2]), as shown in Table 8. After the initiation of the pilot intervention, the number of FP clients decreased monthly at a rate of 252 clients per month. Fig 2 provides a visual display of these results.

**Table 8:** Results of interrupted time series regression, monthly totals of all FP clients in the ten pilot intervention facilities from September 2017 to November 2018, Tanzania 2018.

All FP clients	Coefficient	Std. Err.	t	P>|t|	[95% conf. interval]	
Time (since start of period)	-3.402	26.192	-0.13	0.899	-61.050	54.245
Intervention period	349.197	220.408	2.58	0.141	-135.918	834.312
Interaction of time and intervention period (trend)	-248.598	66.987	-3.71	0.003	-396.035	-101.161
Constant	1687.364	184.356	9.15	0.000	1281.599	2093.128
**Post-intervention linear trend**						
Treated (pilot intervention)	-252.000	61.654	-4.087	0.002	-387.700	-116.301

Regression with Newey-West standard errors. Maximum lag: 0

**Fig 3:**
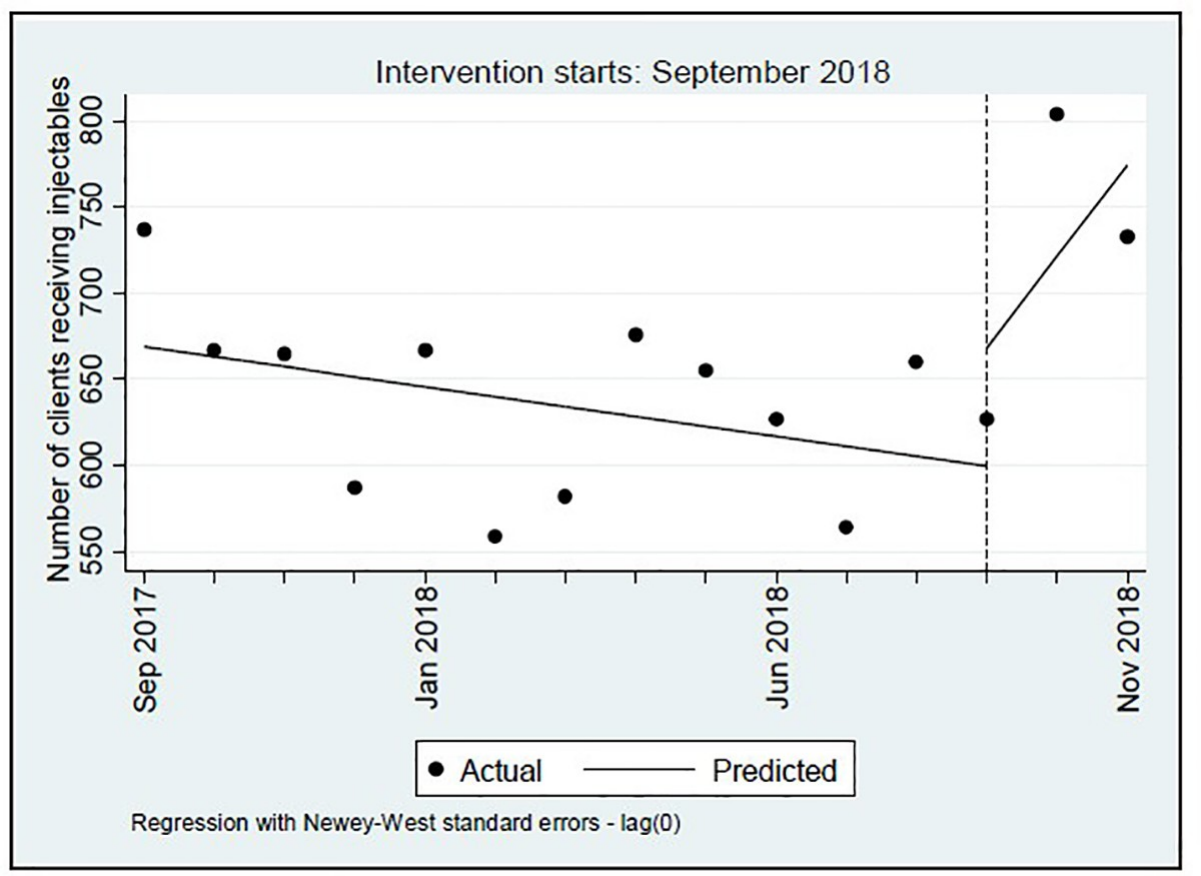
Graph of total number of Depo-Provera clients per month in the ten pilot intervention facilities from September 2017 through November 2018, Tanzania 2018.

Numbers of Depo-Provera clients were also totaled for each month during the 12 months prior to the pilot intervention period and the three months of the intervention. During this 15-month period, the lowest monthly number of clients who received the injectable at the ten facilities was 559 and the highest monthly total was 804; the mean number was 654 [SD = 68.1]. The ITS regression found no statistically significant change in the level or trend of Depo-Provera clients for the intervention period as compared to the pre-intervention period (Table 9). Fig 3 provides a visual display of these results.

**Table 9:** Results of interrupted time series regression, monthly totals of injectable clients in the ten pilot intervention facilities from September 2017 to November 2018, Tanzania 2018.

All FP clients	Coefficient	Std. Err.	t	P>|t|	[95% conf. interval]	
Time (since start of period)	-5.769	4.610	-1.25	0.237	-15.916	4.378
Intervention period	68.667	61.735	1.1	0.290	-67.212	204.545
Interaction of time and intervention period (trend)	58.769	34.440	1.71	0.116	-17.032	134.571
Constant	674.667	34.907	19.33	0.000	597.836	751.497
**Post-intervention linear trend**						
Treated (pilot intervention)	53.000	34.130	-4.087	0.1487	-22.119	128.119

Regression with Newey-West standard errors. Maximum lag: 0

Separate ITS analyses of the monthly number of clients receiving oral contraceptive pills and implants were also conducted. The reported monthly totals of these methods ranged from 102–269 for oral contraceptive pills and 430–941 for implants. Unlike for Depo-Provera, for oral contraceptive pills and implants there was a statistically significant reduction in the number of clients per month during the pilot intervention period as compared to the pre-intervention period (S2 and S3 Tables).

## Discussion

The goal of the evaluation was to provide evidence on the impact of the provision of HC-HIV counseling messages on clients’ knowledge and choice of methods. In August 2019, following analysis of the results of the ECHO Trial, WHO revised the previous categorization of progestogen-only injectable from a “Category 2” to a “Category 1” (i.e. no restrictions) for women at high risk of HIV [12]. Although the evaluation was designed specifically around Depo-Provera and HIV acquisition counseling, the findings are relevant more broadly in FP and HIV programming by providing evidence that complex HIV counseling messages can be implemented in FP programs in Tanzania, and potentially in other countries that are considering how to better integrate HIV risk messages into FP counseling.

Overall, the results indicate that the counseling messages did not cause a decrease in the uptake of Depo-Provera. According to client exit- interviews, the overwhelming majority of women received Depo-Provera at their clinic visit (97 percent) after being given the new HC-HIV counseling messages in addition to usual counseling on the method. Additionally, the analysis of service statistics show no significant difference in the trend of injectable uptake between the pre-intervention and intervention period. This is an important finding, as Depo-Provera is an extremely popular contraceptive method in Tanzania and throughout Sub-Saharan Africa [13].

Dual methods were used by almost 14 percent of clients prior to the clinic visit. After the visit, the reported intention to use condoms to prevent HIV and STIs was high, at 60 percent of interviewed clients (and even higher for adolescents, at 75 percent). Additional analysis of trends in the total number of condoms distributed did not show an increase during the intervention period, suggesting that most clients reporting an intention to use condoms for dual protection did not obtain them from the clinics during this time period [14]. While counseling on dual-method use for women using non-barrier contraceptive methods and who may be at risk of HIV is considered a FP counseling “best practice” [15], evidence from the African region indicates that dual use of methods remains low [16, 17, 18].

Clients’ assessed level of knowledge of HC-HIV messages was highest for messages that are typical of FP counseling sessions—i.e., related to messaging about the effectiveness of hormonal methods to prevent pregnancy and recommendations to use condoms to protect against STIs, including HIV. However, the level of assessed knowledge for the main messages of the new counseling was not as high; additionally, there was confusion about whether other hormonal methods, specifically oral contraceptive pills and implants, also increased the risk of HIV acquisition. Information from the provider interviews may help to explain this finding: the message that other contraceptives do not appear to increase the risk of HIV was mentioned only very rarely by providers as one of the key HC-HIV counseling messages. This may indicate that it was less likely to have been a message that was provided during the counseling session. ITS analysis shows decreasing trends in uptake of other methods during the intervention period. However, these results appear to be unrelated to the provision of the counseling messages, and may in fact be an artifact of the data themselves (as discussed in the limitations section that follows).

In general, few significant differences in outcome measures are found by age group, education level, marital status, or parity. It is possible that the associations were masked by confounding, which was not examined through the bivariate analysis. The results may also be due to the homogeneity of the sample. Almost all interviewed women were educated at the primary, secondary, or higher level (93.6 percent), had one or more living child (97.5 percent), and were married (93.6 percent). Since efforts were made to interview every eligible FP client during data collection, the low level of diversity in these demographic characteristics may reflect the “typical” FP client at these clinics, or perhaps more likely, the “typical” injectable user at these clinics. As a result, generalizations of findings to populations with different characteristics should be made with caution.

Provider’s knowledge of the counseling messages was generally high, especially as assessed by the true/false statements. In spontaneous response, three-fourths of providers mentioned at least three of the HC-HIV counseling messages. These results suggest that there were probably two or three “main” messages that the providers discussed with clients, and that not all of the counseling messages were consistently conveyed.

Providers shared that clients often asked questions immediately after hearing the messages, and that correcting and qualifying the client’s understanding was often part of the counseling session. While providers felt the time spent on providing the HC-HIV counseling messages and answering questions was acceptable, many expressed frustration with having to convey a message based on evidence that was not definitive.

### Limitations

During the provider trainings, the appropriate place in the counseling session to provide the HC-HIV messages was identified as when side effects and other disadvantages of the method were discussed. Because of this placement of the messages, it is likely that clients had already selected Depo-Provera as their method of interest. Thus, the potential effect of the new messaging was not likely on their initial selection of Depo- Provera, but rather, on whether they would stay with the method once they heard the counseling messages. As a result, it is not clear how the messages would affect clients’ initial choice of method.

Women’s perceived HIV risk and variables related to HIV risk, such as number of sex partners, symptoms of STIs, or use of condoms at last sex, were not included in the study. The evaluation did not attempt to identify women who were objectively at “high” risk of HIV acquisition versus women who were not, nor whether women had a correct understanding of their own risk. In fact, research suggests women’s perceptions of HIV risk in high HIV prevalence settings are often inaccurate [19, 20]. Due to the high rate of HIV prevalence in Tanzania, especially in the study regions, all women were considered to be at risk, and were provided the same information during counseling if their HIV status was negative or unknown. However, it is possible that low perceived risk of HIV acquisition in the study population could bias the results if these women were less likely to change their method choice or pay attention to counseling messages. The impact of dual method and HIV risk counseling to women with low perceived risk in high HIV prevalence settings is an area for future study. Additionally, intended condom use compared to actual use after exposure to the counseling messages could not be assessed with this cross-sectional design and is an area for future investigation.

This evaluation design provided an opportunity to test whether the facility-level data were of sufficient quality to be used for ITS analysis. The service statistics tended to “bounce around” over the course of 15 months, and while trend lines could be fitted to the data, there were no clear trends in any direction as observed by the scatterplots either before or during the pilot intervention. The ITS analysis would have benefitted from a pilot intervention period longer than three months (note, a minimum of eight time points after implementation is recommended for ITS [21]). However, this was not feasible given the uncertainty surrounding the effects of the HC-HIV counseling messages. The ITS results should therefore be interpreted with caution. Additional months of the implementation of the HC-HIV counseling messages may have led to some “evening out” of the post-implementation trend lines; thus, findings of significant reductions in the distribution of methods other than Depo-Provera may not be maintained. Furthermore, it is not known the extent to which there were errors in the data recording at the facility level that could have contributed to the wide variability in monthly reports. While ITS is still a recommended analytical tool for answering questions about health services, future analyses using this method in this setting will benefit from continued improvement in the capture of service data and by implementing a longer period of observation in the post-implementation period.

## Conclusions

Results of the evaluation indicate that HC-HIV counseling messages did not cause a decrease in the uptake of Depo-Provera. Furthermore, overall knowledge and understanding of the counseling messages by clients was good, though there was some confusion about whether other hormonal methods, specifically oral contraceptive pills and implants, also potentially increase the risk of HIV acquisition. Providers’ knowledge and comprehension of the HC-HIV counseling messages was high and led to the overall understanding of the intended messages among clients who participated in the pilot intervention.

The evaluation results presented in this report show the potential for successful implementation of complex HIV counseling messages in Tanzania and other countries with high rates of HIV prevalence. Recommendations from this study include clarifying the message that injectables do not contain HIV, and never have, at the beginning of FP/HIV counseling to help disabuse clients of this historical myth and prevent the continuing spread of false information. Another recommendation is to clarify during counseling that there is not an increased risk of HIV acquisition for Depo-Provera compared to other contraceptive methods, as confirmed by the ECHO trial. However, in high HIV prevalence settings, emphasis on dual method use to protect against STIs, including HIV, is essential for all FP users. Finally, client questions stemming from complicated counseling messages, such as side effects or STI/HIV risk assessment, should be anticipated. During counseling, messages may need to be repeated and/or restated in various ways until the client’s questions and concerns are fully addressed.

As countries consider FP and HIV programmatic responses to the ECHO trial results, it is encouraging to have results showing that counseling on complicated HIV messages was able to be delivered and well understood during a pilot intervention. Sustainability issues related to the at-scale provision of new counseling messages include initial and continuing costs of provider training, as well as the production, distribution, and replacement costs of counseling materials. Just as essential, full “buy-in” from healthcare providers will help ensure that messages are provided continuously and consistently to all clients.

## Supporting information

S1 Checklist (PDF)

S1 Table HC-HIV counseling messages. (DOCX)

S2 Table Results of interrupted time series regression, monthly totals of oral contraceptive pill clients in the ten pilot intervention facilities from September 2017 to November 2018, Tanzania 2018. (DOCX)

S3 Table Results of interrupted time series regression, monthly totals of implant clients in the ten pilot intervention facilities from September 2017 to November 2018, Tanzania 2018. (DOCX)

S1 File (PDF)
