# Supplementary material for: Counseling on injectable contraception and HIV risk: Evaluation of a pilot intervention in Tanzania
Source: PLoS One. 2020 Apr 3;15(4):e0231070. doi: 10.1371/journal.pone.0231070 (PMC7122807; doi:10.1371/journal.pone.0231070)
Supplement: S1 Table — (DOCX) [file pone.0231070.s002.docx]

# S1 Table. HC-HIV counselling messages

| For all women | - Hormonal contraceptives are extremely effective in preventing unintended pregnancies when used consistently and correctly. - Hormonal contraception will not protect you from sexually transmitted infections (STIs), including HIV. It is important to also use a male or female condom with hormonal contraceptive to ensure dual protection from both STIs and pregnancy. - If you are not in a mutually monogamous relationship, it is important to use condoms in addition to hormonal contraception. - All women regardless of their HIV status can use hormonal contraception. |
| --- | --- |
| For women who are HIV negative or of unknown HIV status who choose to use Depo-Provera | - The World Health Organization approves the use of Depo-Provera for all women, including women at high risk of HIV, but recommends that women who chose Depo-Provera be counselled on the possible increased risk for HIV and ways to prevent HIV transmission. - You can protect yourself from HIV by using condoms consistently and correctly- in addition to Depo-Provera or another hormonal contraceptive method. - You can use any method you would like and can still choose to use Depo-Provera if that is the method you prefer. - There are other effective family planning methods to choose from if you decide that you do not want to start Depo-Provera or if you want to switch to a different method. |
