# Supplementary material for: Counseling on injectable contraception and HIV risk: Evaluation of a pilot intervention in Tanzania
Source: PLoS One. 2020 Apr 3;15(4):e0231070. doi: 10.1371/journal.pone.0231070 (PMC7122807; doi:10.1371/journal.pone.0231070)
Supplement: S2 Table — (DOCX) [file pone.0231070.s003.docx]

**S2 Table. Results of interrupted time series regression, monthly totals of oral contraceptive pill clients in the ten pilot intervention facilities from September 2017 to November 2018, Tanzania 2018**

| **All FP clients** | **Coefficient** | **Std. Err.** | **t** | **P>\|t\|** | **[95% conf. interval]** | |
| --- | --- | --- | --- | --- | --- | --- |
| Time (since start of period) | -0.990 | 4.638 | -0.21 | 0.835 | -11.196 | 9.217 |
| Intervention period | 34.348 | 27.617 | 1.24 | 0.239 | -26.437 | 95.134 |
| Interaction of time and intervention period (trend) | -23.010 | 8.298 | -2.77 | 0.018 | -41.274 | -4.747 |
| Constant | 185.182 | 41.581 | 4.45 | 0.001 | 93.662 | 276.702 |
| **Post-intervention linear trend** | | | | | | |
| Treated (pilot intervention) | -24.000 | 6.881 | -3.488 | 0.005 | -39.145 | -8.855 |

Note: Regression with Newey-West standard errors. Maximum lag: 0
