# Supplementary material for: Counseling on injectable contraception and HIV risk: Evaluation of a pilot intervention in Tanzania
Source: PLoS One. 2020 Apr 3;15(4):e0231070. doi: 10.1371/journal.pone.0231070 (PMC7122807; doi:10.1371/journal.pone.0231070)
Supplement: S3 Table — (DOCX) [file pone.0231070.s004.docx]

**S3 Table. Results of interrupted time series regression, monthly totals of implant clients in the ten pilot intervention facilities from September 2017 to November 2018, Tanzania 2018**

| **All FP clients** | **Coefficient** | **Std. Err.** | **t** | **P>\|t\|** | **[95% conf. interval]** | |
| --- | --- | --- | --- | --- | --- | --- |
| Time (since start of period) | 0.972 | 15.312 | 0.06 | 0.951 | -32.729 | 34.673 |
| Intervention period | 178.682 | 143.300 | 1.25 | 0.238 | -136.719 | 494.083 |
| Interaction of time and intervention period (trend) | -173.472 | 57.536 | -3.02 | 0.012 | -300.108 | -46.837 |
| Constant | 672.515 | 106.948 | 6.29 | 0.000 | 437.124 | 907.907 |
| **Post-intervention linear trend** | | | | | | |
| Treated (pilot intervention) | -172.500 | 55.461 | -3.110 | 0.010 | -294.569 | -5.431 |

Note: Regression with Newey-West standard errors. Maximum lag: 0
