# Supplementary material for: Counseling on injectable contraception and HIV risk: Evaluation of a pilot intervention in Tanzania
Source: PLoS One. 2020 Apr 3;15(4):e0231070. doi: 10.1371/journal.pone.0231070 (PMC7122807; doi:10.1371/journal.pone.0231070)
Supplement: S1 File — (PDF) [file pone.0231070.s005.pdf]

Study Protocol

Submitted: April 19, 2018

Approved by University of North Carolina at Chapel Hill IRB #18-1049

Title

Impact of HIV Risk Communication on Hormonal Contraceptive Uptake in Tanzania

Brief Summary

Purpose: The World Health Organization recently updated its guidance on hormonal contraceptive use by women who are at high risk of HIV, based on observational evidence that suggests progesterone-only injectables may increase risk of HIV acquisition. The Ministry of Health in Tanzania is piloting new family planning counseling messages that incorporate the updated guidance in ten health care facilities. This evaluation will assess client's understanding of the new counseling messages and the impact of the counseling on the initiation and continuation of progesterone-only hormonal injectables, other hormonal methods, and dual method use. Participants: 450 female family planning clients and up to 20 health service providers Procedures (methods): The evaluation will use a mixed methods approach and include client exit interviews, provider interviews, and a time-series design to track trends in uptake of the above mentioned family planning methods using aggregated service statistics.

Principal Investigator: Barden-O'Fallon, Janine
Department: Carolina Population Center

Funding Source

Agency for International Development (USAID)

Scientific Review

This protocol does not involve interaction or intervention that poses greater than minimal risk to subjects. Minimal Risk-the probability and magnitude of harm or discomfort anticipated in the research are not greater than those ordinarily encountered in daily life or during the performance of routine physical or psychological examinations or tests.

Background and Rationale

The World Health Organization (WHO) recently updated its guidance on hormonal contraceptive (HC) use by women who are at high risk of HIV, based on recent evidence that suggests progesterone-only injectables may increase risk of HIV acquisition. The Ministry of Health in Tanzania is piloting new family planning (FP) counseling messages that incorporate the updated guidance. The counseling messages are being implemented in ten facilities located in two regions of Tanzania over a 3.5-month period in 2018. The evaluation will use facility-level routine service statistics on contraceptive uptake before and

throughout the implementation of the counseling intervention to assess whether the messages result in changes in contraceptive uptake, including method mix. Clients will also be interviewed to assess their level of understanding of the messages and the degree to which the messages influence their contraceptive decision-making. Providers will be interviewed to assess their comfort with the messaging and their perception of clients' understanding. These data will be combined with monitoring data on quality of counseling throughout the intervention to provide a comprehensive assessment of the effects of implementing the new counseling messages. Evidence from the proposed HC-HIV communications activity will be ground-breaking, with the potential to influence FP programming efforts globally, especially FP counseling, FP method mix and HIV prevention efforts. It will ultimately help to improve the quality of FP programming in Tanzania. It will also help to strengthen FP and HIV counseling efforts and improve provider-client relationships.

Research questions

1. To what extent do new counseling messages change the uptake of Depo Provera among FP clients exposed to the messages? This will be measured in both potential new users and continuing users of Depo Provera.
2. To what extent do new counseling messages change the uptake of other hormonal methods, such as oral contraceptive pills and implants, among FP clients exposed to the messages?
3. To what extent do new counseling messages influence the reported use or intention to use condoms among FP clients exposed to the messages? Condom use includes single-method and dual-method use.
4. To what extent do FP clients have correct knowledge of the new counseling messages? What do FP clients believe about the potential increased risk of HIV acquisition? Are there differences in the level of knowledge by age, marital status or education level?
5. How do healthcare providers respond to the new counseling messages? What are their attitudes about their own ability to correctly communicate the messages? What, if any, concerns do healthcare providers have about the messages?

Additionally, the provision of the counseling messages by trained healthcare providers will be monitored by provider observations throughout the 3.5-month implementation to determine the degree to which healthcare providers maintain fidelity to the counseling messages

Evaluation design

General design: The evaluation will assess the effects of new communication messages on uptake of Depo Provera and hormonal contraceptive methods, reported use of condoms as a single or dual use method, and the perceptions of female FP clients and FP healthcare providers on the counseling messages. The evaluation will use a mixed methods approach and include multiple types of data: client exit interviews, provider interviews, routine service statistics, and routine monitoring of provider-client counseling to provide a comprehensive understanding of the impact of the HC-HIV counseling messages. Specifically, client's understanding of the communication messages, intention to use condoms, and FP method chosen during their appointment will be assessed through a short client exit interview, utilizing both closed and open-ended questions. Provider's feedback on the messages and their level of comfort

providing the messages will be assessed through key informant interviews (KIIs). A time-series design to track overall trends in uptake of Depo Provera, other hormonal methods, and condoms distributed, before and after the intervention will be undertaken with data from health facility FP service statistics.

Study area

The intervention will be piloted in two regions of Tanzania; Iringa and Njombe. These regions were selected due to the relatively high level of modern contraceptive use (32% in Iringa and 45% in Njombe) and HIV prevalence (11.3% in Iringa and 11.4% in Njombe). Five primary health care facilities with high numbers of Depo Provera (the most common progesterone-only injectable uses in Tanzania) uptake from March 2017 to February 2018 were selected as intervention sites in each region. Among the ten selected facilities, annual Depo Provera uptake ranged from 1,933 clients at Njombe Health Center to 414 clients at Ihongole Health Center.

Selected health facilities from Iringa are Mafinga Hospital, Ilula Hospital, Ipogolo Health Center, Kimande Health Center and Ihongole Health Center. Selected health facilities from Njombe are Njombe Health Center, Njombe District Hospital, Makambako Hospital, Ipelele Health Center and Ihongole Health Center. All the FP service providers from the selected health facilities will receive training and counseling materials to participate in the intervention. It is anticipated that each selected health facility will have 3-5 FP service providers, thus the total number of trained providers will be between 30-50. Women attending these health facilities during the intervention period who are currently using Depo Provera or express interest in using Depo Provera as the FP method of choice will be eligible to receive counseling on the risk of HIV acquisition.

Client exit interviews

Exit interviews will be conducted with women exposed to the counseling message. Women will be eligible for a client exit interview if they 1) attend a FP appointment at one of the ten healthcare facilities involved in the intervention during the period of data collection and 2) receive the counseling messages. Eligible women include continuing Depo Provera users and new Depo Provera clients, as well as women who received the counseling messages once selecting Depo Provera as their method of choice and then selected an alternative method. There is no minimum or maximum age restriction, though it is assumed that all women will be of reproductive age (approximately ages 15-49). Women will be approached for client exit interviews at random, though an attempt will be made to ensure that clients from all trained FP providers at the facility are approached for interviews. Information on women's understanding of the messages will be assessed through a short interview with closed and open-ended questions. Decision-making about contraceptive use during their FP appointment will be included, as well as method used when coming to the appointment, method accepted at the appointment, and planned condom use. Planned condom use will be self-reported, with the caveat that intentions may not represent actual future behavior. Actual condom use after the exposure to the counseling messages or longer-term outcomes such as changes in contraceptive methods that happen after the index visit to the healthcare facility (particularly for continuing use of Depo Provera), cannot be assessed with this cross-sectional design. Pending results of the current evaluation study, such issues may be worthy of future investigation. In addition to information on the outcomes of interest (i.e.

correct knowledge of the counseling messages, contraceptive decision-making, and current and planned condom use), demographic information that will be collected in client exit interviews include age of clients (ages 15-24 “youth” vs ages 25-49 “adult”), marital status (married/in union vs unmarried/single), number of children, and education level (none, primary only, secondary+).

Provider interviews

Key informant interviews will be conducted with 10-20 healthcare providers (1-2 providers from each of the participating facilities). Healthcare providers will be randomly selected for interview at each facility among eligible providers. Eligible providers are those who 1) received the training on new HC-HIV counseling messages, 2) participated in the intervention, and 3) are available for the interview on the day of data collection. If no providers are available for interview on the day of data collection, the data collection team will make arrangements to return so that at least one provider is interviewed at every healthcare facility. Healthcare providers will be asked about their perceptions of the counseling messages; whether they feel women understand the messaging, whether they feel the counseling materials are useful, what types of questions and concerns are being expressed by clients, and how comfortable they feel providing the counseling message. Providers will be asked if the counseling messages and/or tools should be improved, and ways in which the intervention could be improved for future roll out. The provider interviews will be recorded since the interview includes several open-ended questions.

Time Series Analysis

In addition to information on contraceptive use collected through client exit interviews, the evaluation will use interrupted time series (ITS) analysis using segmented regression to assess the overall impact of the counseling messages on contraceptive use among FP clientele in participating health facilities. ITS is a quasi-experimental evaluation method that has been used for a wide range of public health interventions (recent examples include Delamou et al., 2017; Iyer et al., 2017; Singh et al., 2016). In this method, a ‘time series’ is a continuous sequence of observations, taken repeatedly (at equal intervals) over time. In an ITS study, a time series of a particular outcome of interest, in this case Depo Provera uptake, is used to establish an underlying trend, which is ‘interrupted’ by the intervention at a known point in time. The hypothetical scenario under which the intervention had not taken place and the trend continues unchanged (that is: the ‘expected’ trend, in the absence of the intervention, given the pre-existing trend) is referred to as the ‘counterfactual’ (Bernal, Cummins, Gasparrini, 2017). This counterfactual scenario provides a comparison for the evaluation of the impact of the intervention by examining any change occurring in the post-intervention period. In this way, causal effects can be estimated with an observational approach, making this a “next best” option to a randomized design (Kontopantelis et al., 2015).

A minimum of three variables are required for an ITS analysis:

1. T: the time elapsed since the start of the study period, in months
2. X_t : a dummy variable indicating the pre-intervention period (coded 0) or the post-intervention period (coded 1);

3. Y_t 3. : the outcome at time t

In standard ITS analyses, the following segmented regression model is used:

$$Y_t=\beta_0 + \beta_1 T + \beta_2 X_t + \beta_3 T X_t$$

where β_0 represents the baseline level at T = 0, β_1 is interpreted as the change in outcome associated with a time unit increase (representing the underlying pre-intervention trend), β_2 is the level change following the intervention and β_3 indicates the slope change following the intervention (using the interaction between time and intervention: TXt) (Bernal, Cummins, Gasparrini, 2017).

Monthly totals of contraceptive uptake by method from each facility participating in the intervention will be collected from one year prior to the initiation of the intervention through the completion of the intervention. Thus, there will be a total of 16 months, 12.5 prior to the intervention and 3.5 of the intervention. Monthly facility totals for each method will be pooled for analysis; analysis of individual facilities will be attempted if there is sufficient power to obtain valid estimates. The selection of ITS as a method for this evaluation is supported by the fact that there is a clear, defined initiation of the intervention; the outcomes are well-defined and 'immediate' (i.e., the selection of methods happens at the facility and is recorded); routine data are available; and (sequential) measures of the outcome will be collected from facility records from before and after the intervention. This analysis offers an opportunity to work with a source of underutilized data (i.e., routine service statistics) and promote strengthening of routine data systems by working with stakeholders at the facility, regional, and national level, to use the data to answer clinically relevant questions.

The interpretation of the ITS model will be made in conjunction with information collected from clients and providers (as described above). The expectation of the ITS model is that there will be no change in the gradient of the trend for contraceptive use over the course of the intervention. In other words, if the general trend is for increasing numbers of clients initiating contraception over time, and the intervention is successful so that the uptake of contraception is unaffected by the new counseling messages and clients correctly understand the risk messages, the trend of increasing contraceptive use over time would continue throughout the period of the intervention. This result may comprise of declines in particular methods, such as Depo Provera, but compensating increases in the uptake of other methods, such as pills or implants. Thus, the analysis and interpretation of trends will be made for all contraceptives and by method. In addition, if the intervention is successful and clients correctly understand the risk messages, an increase in condom use (as assessed by the registers) as a dual method may be observed after exposure to the counseling messages.

The ITS design is typically unaffected by confounders, however, it does not fully control for time varying factors that may affect contraceptive use trends, such as trends that could influence the use of HC but are unrelated to the counseling messages. Any change in data collection related to method uptake in the intervention facilities during the intervention could also bias results. Another potential threat to validity is seasonality, however, seasonal patterns of use will be controlled for as the period of data will cover more than one full year and data from the season of intervention will be collected in the pre-intervention period. Another potential threat to the validity of findings would be a small effect of the

intervention on method uptake. In such a case, the findings would have large error terms and would need to be interpreted with caution. Finally, high quality routine data at the facility level, including accuracy and completeness, is necessary to attain valid results. This evaluation design will test whether the facility-level data is of sufficient quality to be used for such an analysis. A case for maintaining and/or strengthening data quality at the facility level can be made to stakeholders, especially health care providers, pending the findings. Use of service statistics as a means for answering questions about health services, and the effects of changes in any element of health service provision, can be emphasized.

Monitoring the intervention

Monitoring the provision of the counseling is essential to determine if healthcare providers are following through with the counseling messages and if they are delivering them as they were trained to do. In this way, adherence to the intervention as it was designed will be assessed and will provide important information to the implementation and evaluation teams. The implementing partner responsible for the intervention has incorporated quality assurance monitoring into the implementation of the intervention. The study team will work closely with this implementing partner to ensure appropriate monitoring data are collected.

Data collection, analysis and disposition

Clients and providers will be interviewed face-to-face, in Swahili or English. Both client and provider interview guides include closed and open-ended questions. Monthly FP service statistics will be abstracted from facility registers using a data abstraction form. The data will be collected using "paper and pencil" and entered into an electronic database. While still in the field, the data collection team will be responsible for entering the data into a computer, using a software package such as CSPro, and running initial quality checks. All data will be kept on password protected computers, with access limited to members of the study team. Data will be transferred to MEASURE Evaluation using the Secure File Transfer Protocol (SFTP). Copies of paper surveys will be kept in a locked cabinet until the analysis of the data is complete, at which point, they will be destroyed. After the completion of analysis, the data collection team will also scrub the data from their computer(s).

Overall descriptive statistics of the interviewed clients will be generated for outcomes and demographic indicators of interest. Outcome indicators of correct knowledge, contraceptive decision-making, and change in contraceptive use will be assessed overall, by region, by facility, and by individual-level demographic variables. The relationship between outcome indicators and individual, facility, and region variables will be assessed through regression analysis. Correlation of observations within facilities will be controlled using econometric techniques. Regression models will be run for the time series analysis. A p-value of 0.05 will be used to assign statistical significance. STATA version 14.2 will be used for the analysis. Qualitative data coming from open-ended questions in the interviews will be translated into English, if needed, before analysis. A qualitative software package, such as NVivo, will be used to code for themes emerging from the interviews. Coding categories will be comprised of discrete comments, thoughts, opinions, or ideas on a particular topic. Quotes that represent emergent themes will also be identified.

Ethical considerations

The proposal and draft data collection tools will be submitted to the University of North Carolina Institutional Review Board (UNC IRB) and the Tanzania national ethics board (National Institute for Medical Research, NIMR). Research clearance will also be sought from the Tanzania Commission for Science and Technology (COSTECH), which is the government's research clearinghouse. The research activities will not begin prior to the approval of the UNC IRB, NIMR, and COSTECH.

Client exit interviews and provider key informant interviews are planned to occur between July and September 2018. Extraction of routine health data at facilities is planned to occur in October. No additional data collection will occur thereafter. The study is expected to end no later than March 2019.

The data collection team will undergo a 1-week training on the the client and provider interviewer guides and data extraction tool for routine facility data. The training will include a pretest of the tools and procedures before team members begin actual data collection. Additionally, all team members will receive training on ethics.

Benefits to subjects and/or society

Evidence from the proposed HC-HIV communications activity will be ground-breaking, with the potential to influence FP programming efforts globally, especially FP counseling, FP method mix and HIV prevention efforts. It will ultimately help to improve the quality of FP programming in Tanzania. It will also help to strengthen FP and HIV counseling efforts and improve provider-client relationships.

There is no direct benefit to subjects for their participation in the study. However, clients may benefit if they are recipients of improved FP counseling in the future.

Risks and measures to minimize risk

We expect that there will be minimal risk of emotional distress, embarrassment or breach of confidentiality, if any at all. The client exit interview includes questions about clients' FP behaviors, which may be an uncomfortable or embarrassing subject for some respondents. However, the data collection team will receive extensive training on the tools and ethical issues, including rapport-building and how to deal with sensitive questions. Psychological or emotional distress may ensue from a breach of confidentiality. We anticipate the likelihood of such a breach to be rare. The training on ethical conduct of research will emphasize strict protection of confidentiality. The data collection team will be required to sign a document certifying their commitment to maintaining participant confidentiality. Hard copy instruments will not include the participants' names, and the interview will be conducted privately to ensure confidentiality.

Clients: we anticipate very minimal potential for deductive disclosure.

Providers: potential deductive disclosure is possible for providers, because there are few providers per facility. However, the provider interview does not include sensitive information and primarily focuses on their perceptions and understanding of the HC-HIV counseling messages.

Subjects

Total number of subjects proposed across all sites by all investigators is 470. This includes 450 women of reproductive age who attended a FP appointment at one of the selected health facilities during data collection and received the new HC-HIV counseling messages and 20 healthcare providers who work at one of the selected health facilities, received training on the new HC-HIV counseling messages, and are participating in the intervention.

The tools and consent forms will be available in Swahili and English to reflect the official languages of Tanzania.

Inclusion of children (under age of majority for their location):

Women of reproductive age (15-49 years) who received FP services at the study sites will be asked for their participation in the study. The study does NOT target recruitment of children (under 18 years), but can include women as young as 15 years of age. Participants who are ages 15-17 will not require parental consent for their participation. Some of these individuals may be sexually active and using FP methods without their parent's knowledge, thus requesting parental consent could place them at additional risk due to the disclosure of sexual activity. Additionally, in Tanzania the median age at marriage is 19.2 years, so it is likely that many of these women would be married and considered emancipated minors.

Women ages 15-17 years will be asked to sign an assent form before their participation in the study. A waiver of parental permission is requested for women ages 15-17 years.

There is no benefit specific to the participants who are ages 15-17. However, since women in this age group use FP methods and will be exposed to the new counseling messages, it is important to know if they understand the messages to the same extent as women in other age groups. There are no unique risks associated with the participants who are ages 15-17.

Inclusion/Exclusion criteria:

Client exit interviews: Women will be eligible for a client exit interview if they 1) attend a FP appointment at one of the ten healthcare facilities involved in the intervention during the period of data collection and 2) receive the counseling messages. Eligible women include continuing Depo Provera users and new Depo Provera clients, as well as women who received the counseling messages once selecting Depo Provera as their method of choice and then selected an alternative method.

Provider key informant interviews: Providers will be eligible if they 1) received training on the new counseling messages, 2) participated in the intervention, and 3) are available for interview on the day of data collection.

Men are excluded from the client exit interviews, because men do not receive Depo Provera for FP.

Sample size

The sample size was determined using an acceptable level of significance of $p < 0.05$, a power of 80%, and a population size of 150,000 (the population is defined as sexually active women of reproductive

age living in the catchment areas of the ten intervention facilities, and is an estimate). The calculated sample size is 383; an additional 10% is added to the sample size to account for incomplete surveys and other potential data quality issues (n=422), and then rounded up to 450 to take advantage of as much data as possible. According to DHIS 2 data for the 12 month period of March 2017 to February 2018, the number of Depo Provera clients seen per week ranges from 8-37 clients in the selected facilities of Iringa and Njombe. An average of 45 FP clients will be interviewed at each of the 10 facilities, though the actual number will vary according to client volume. The final number of interviews per facility will be determined during training for data collection.

Providers for key informant interviews

Key informant interviews will be conducted with 10-20 healthcare providers (1-2 providers from each of the participating facilities).

Data and safety monitoring

Data collection teams will include trained interviewers and trained supervisors. All research staff will be trained on consent procedures and confidentiality and proper interview techniques with the client interview guide. Participants may refuse to participate in the interview at any time. No data (hard or soft copy) will have personal identifiers. Signatures will be collected on consent forms only. Names will not be linked to interviews. Consent forms will be kept in a locked cabinet and kept separate from hard-copies of completed interviews. All data collection forms will be reviewed by the team supervisor for quality assurance. Hard copy forms will be stored in a secure location and electronic data stored on secure and password-protected servers.

To ensure data protection and confidentiality across the study, all team members will commit to using reasonable data protection measures to protect the data. Data collection strategies will be put in place to maintain a high level of confidentiality. Confidentiality will be discussed in detail with the data collection team during training session on research ethics. The principal investigator and the study team will conduct ongoing review of study processes throughout the data collection period. A member of the supervisory team from UNC will accompany the interview team for the first week of data collection to ensure that the protocol is being appropriately followed and to ensure participant rights and safety. Field supervisors from the data collection team will review questionnaires and other data collection forms on a daily basis and will provide feedback to interviewers if deviations in the protocol or questionnaire completion have occurred. Field supervisors will also be required to complete weekly field report forms documenting any emergent problems that arise during interactions with participants. The initial training of the field teams will identify potential situations or types of situations that would require immediate notification by phone to the UNC supervisory team, such as a breach of confidentiality.

Client and provider interviews will not contain names or other explicit identifiers. Routine data extracted from the facility are aggregated data that do not include any identifiers, e.g. the total number of women that received oral contraceptives at the facility. Nevertheless, all hard copy data will be kept in a locked and secured cabinet until after the conclusion of data analysis, at which point they will be destroyed. Electronic data will be stored in a password-protected and encrypted computer database at the Carolina

Population Center, University of North Carolina at Chapel Hill. All data will only be accessed by authorized members of the study team.

A local consultant or group will be contracted to manage and implement data collection. There are no identifiers collected as part of the study. However, consent forms (containing signatures) will be collected by trained research staff and handed in-person to a supervisor, who will store in a locked cabinet and in a secure location. All research staff will be trained on research ethics principals, including confidentiality. Consent forms (containing signatures) will be collected by trained research staff and handed in-person to a supervisor, who will store the forms in a locked cabinet and in a secure location.

Data analysis

Quantitative data

Overall descriptive statistics of the interviewed clients will be generated for outcomes and demographic indicators of interest. Outcome indicators of correct knowledge, contraceptive decision-making, and change in contraceptive use will be assessed overall, by region, by facility, and by individual-level demographic variables. The relationship between outcome indicators and individual, facility, and region variables will be assessed through regression analysis. Correlation of observations within facilities will be controlled using econometric techniques. Regression models will be run for the time series analysis. A p-value of 0.05 will be used to assign statistical significance. STATA version 14.2 will be used for the analysis.

Qualitative data

A qualitative software package, such as NVivo, may be used to code for themes emerging from the interviews. Coding categories will be comprised of discrete comments, thoughts, opinions, or ideas on a particular topic. Quotes that represent emergent themes will also be identified. Information resulting from the analysis of client interviews, provider interviews, service statistics, and monitoring data, will be triangulated in order to generate a comprehensive report on the effects of the counseling intervention.

Direct interaction

Women will be approached for client exit interviews at random, after the completion of their FP clinic visit. Approximately two providers will be randomly selected and interviewed at each of the 10 selected study sites.

Healthcare facilities in Tanzania have designated areas for FP services. The data collection team will be positioned in the FP areas and interview women completing their FP clinic visits. An average of 45 FP clients will be interviewed at each of the 10 facilities, though the actual number will vary according to client volume.

Healthcare providers from the study facilities will be informed of the evaluation during their training. Approximately two providers will be randomly selected and interviewed at each of the 10 selected study sites, with a minimum of 1 per participating facility.

The research staff will work with the facility in-charge to ensure that the location where clients will be recruited and interviewed is a private space to ensure confidentiality. For example, the area will be a space where the respondent's answers cannot be heard by others.

Number of contacts per subject = 1

Duration of contact = approximately 1 hour

Existing data

FP service data is routinely collected at facilities on nationally standardized registers, then aggregated and summarized on standard reporting forms. For this study, monthly FP service statistics will be abstracted from the standard reporting form, using a data abstraction tool.

Access to the health facilities, to conduct interviews and data extraction, require national and subnational levels of approvals. The Tanzania Ministry of Health is providing leadership and support for the intervention and evaluation, and relevant MOH personnel will help to facilitate necessary approvals.

Consent and assent process

After initial contact, trained research staff will provide the subject with a written consent or assent form, explain the study and go over the content of the form. The form will be available in English and in Swahili. The form will detail the study procedures including potential risks and benefits, confidentiality and time spent participating in the research. Potential participants will also be told that they are free at all times not to participate in the research and that refusal to participate will in no way affect future services they receive at the health facility. Participants will read or have read to them the consent or assent form, and be encouraged to share with research staff any concern they have regarding their participation. Written informed consent or assent will be obtained prior to the initiation of the client exit interview. Participants who are unable to read or sign their name can make a mark (a cross) in the presence of a witness. Signed copies of the form will be given to the participants.

Provider key informant interviews: Providers will undergo a similar consenting process and sign a consent form before being interviewed. Non-participation will not affect their employment at the facility.

Research staff who are fluent in Swahili and English will be recruited and trained to administer the consent process and interviews. The consent and assent forms will be available in Swahili and English. The interview will ask potential participants their preferred language, and proceed with the consent and assent process in that language. Research staff may include collaborators from the Ministry of Health as well as the trained data collectors.
